# Clinical significance of the preoperative platelet count and platelet-to-lymphocyte ratio (PLT-PLR) in patients with surgically resected non-small cell lung cancer

**DOI:** 10.18632/oncotarget.8809

**Published:** 2016-04-19

**Authors:** Seok-Hyun Kim, Hyoun Wook Lee, Se-Il Go, Soon Il Lee, Gyeong-Won Lee

**Affiliations:** ^1^ Division of Hematology and Medical Oncology, Department of Internal Medicine, Samsung Changwon Hospital, Sungkyunkwan University School of Medicine, Changwon, Republic of Korea; ^2^ Department of Pathology, Samsung Changwon Hospital, Sungkyunkwan University School of Medicine, Changwon, Republic of Korea; ^3^ ivision of Hematology-Oncology, Department of Internal Medicine, Gyeongsang National University Changwon Hospital, Changwon, Republic of Korea; ^4^ Division of Hematology-Oncology, Department of Internal Medicine, Gyeongsang National University Hospital, Gyeongsang National University School of Medicine, Jinju, Republic of Korea; ^5^ Department of Internal Medicine, Dankook University College of Medicine, Cheonan, Republic of Korea; ^6^ Gyeongsang Institute of Health Sciences, Gyeongsang National University School of Medicine, Jinju, Republic of Korea

**Keywords:** non-small cell lung cancer, platelet-to-lymphocyte ratio, thrombocytosis, inflammation, prognosis

## Abstract

**Background:**

The aim of this study was to assess the prognostic significance of the preoperative platelet count (PLT) and platelet-to-lymphocyte ratio (PLR) in patients with surgically resected non-small-cell lung cancer (NSCLC).

**Patients and Methods:**

We retrospectively reviewed 202 patients treated for NSCLC between January 2002 and December 2007. Preoperative PLT and PLR scores were calculated using data obtained at the time of admission. Patients were assigned a PLT-PLR score of 0, 1, or 2 based upon the presence of thrombocytosis, an elevated PLR, or both.

**Results:**

Patients with a PLT-PLR score of 2 had a significantly lower median overall survival (OS) [12.715 mo; 95% confidence interval (CI) 1.215-24.215] when compared with patients with PLT-PLR scores of 1 (52.238 mo; 95% CI 17.062-87.414, *p* = 0.002) or 0 (not reached, *p* < 0.001). Relapse-free survival (RFS) was also significantly decreased in patients with a PLT-PLR score of 2 (10.107 mo; 95% CI 3.388-16.826) relative to patients with a PLT-PLR score of 1 (27.214 mo; 95% CI 0-56.253, *p* = 0.002) or 0 (58.893 mo; 95% CI 32.938-84.848, *p* < 0.001). In multivariate analysis, a PLT-PLR score of 2 was an independent prognostic factor for poor OS (hazard ratio (HR) 3.473; 95% CI 1.765-6.835, *p* < 0.001) and RFS (HR 2.286; 95% CI 1.243-4.206, *p* = 0.008) compared with a PLT-PLR score of 0.

**Conclusions:**

Preoperative PLT-PLR scores can be useful for predicting disease prognosis in patients with surgically resected NSCLC. Further large prospective studies will be necessary to validate our findings.

## INTRODUCTION

Lung cancer is the leading cause of cancer-related mortality worldwide [1]. Non-small cell lung cancer (NSCLC) accounts for approximately 85% of lung cancer cases [2]. Among NSCLC patients with resectable disease, the prognosis is dependent on the disease stage, with 5-year overall survival (OS) rates ranging from 73% for stage IA to 24% for stage IIIA disease [3]. Even in patients with pathologic stage I disease, for whom adjuvant therapy is not considered, the 5-year OS rates vary from 57% to 85% and are not satisfactory for indiscriminate exclusion of these patients from adjuvant therapy [4–6]. Therefore, a new biomarker to predict surgical outcomes is needed to identify patients with localized NSCLC who will benefit from adjuvant therapy.

Recent studies have demonstrated a significant role of platelets during cancer progression and metastases. Complex interactions among platelets, tumor cells, and the tumor microenvironment result in tumor growth, aberrant angiogenesis, invasion, and metastasis [7, 8]. Furthermore, reactive or paraneoplastic thrombocytosis is commonly induced by inflammation and abnormal release of cytokines in the interactive response of the host to cancer [8]. The platelet-to-lymphocyte ratio (PLR) [9–12] and thrombocytosis [13–15] have been assessed to evaluate the association between platelets and cancer progression. However, there is no consensus as to which is a more reliable marker predicting the prognosis of cancer patients.

In this study, we incorporated both markers into what we referred to as the PLT-PLR (platelet count and platelet-to-lymphocyte count ratio) score. Then, the role of the PLT-PLR score as a prognostic marker was assessed in patients with surgically resectable NSCLC.

## RESULTS

### Patient characteristics

This study included 202 patients with histologically confirmed, resected NSCLC. Of these, 169 (83.7%) were male, with a median age at diagnosis of 64 years (range, 31–77 years). The most prevalent histology was squamous cell carcinoma (SqCC) (125/202, 61.9%). The majority of patients (165/202, 81.0%) had stage I or II disease, and adjuvant treatment was performed in only 57 patients (57/202, 28.2%). A full list of patient characteristics based on the PLT-PLR score is shown in Table 1. No statistically significant differences in terms of patient characteristics were detected between groups, with the exception of the type of surgery and T classification.

**Table 1:** Patients' characteristics according to PLT-PLR

Variables	Group	N	PLT-PLR			*P*
			0 (n=108, %)	1 (n=79, %)	2 (n=15, %)	
**Sex**	Male Female	169 33	88(81.5) 20(18.5)	67(84.8) 12(15.2)	14(93.3) 1(6.7)	0.447
**Age (yr)**	< 65 ≥ 65	91 111	50(46.3) 58(53.7)	34(43.0) 45(57.0)	7(46.7) 8(53.3)	0.899
**Smoking**	Never smoker Current or former smoker	68 134	34(31.5) 74(68.5)	32(40.5) 47(59.5)	2(13.3) 13(86.7)	0.097
**Histology**	SqCC Non-SqCC^a^	125 77	61(56.5) 47(43.5)	53(67.1) 26(32.9)	11(73.3) 4(26.7)	0.215
**ECOG PS**	0 1	136 66	74(68.5) 34(31.5)	52(65.8) 27(34.2)	10(66.7) 5(33.3)	0.926
**Surgery**	Lobectomy Pneumonectomy Bilobectomy or sleeve op. Wedge resection Segmentectomy	163 30 6 2 1	95(88.0) 9(8.3) 3(2.8) 0(0) 1(0.9)	60(75.9) 16(20.3) 2(2.5) 1(1.3) 0(0)	8(53.3) 5(33.3) 1(6.7) 1(6.7) 0(0)	0.021*
**T classification**	T1a to T2b T3 to T4	178 24	98(90.7) 10(9.3)	70(88.6) 9(11.4)	10(66.7) 5(33.3)	0.026
**N classification**	N0 to N1 N2 to N3	183 19	94(87.0) 14(13.0)	75(94.9) 4(5.1)	14(93.3) 1(6.7)	0.175
**TNM stage**	pI pII pIII	95 70 37	54 (50.0) 35 (32.4) 19 (17.6)	38 (48.1) 29 (36.7) 12 (15.2)	3 (20.0) 6 (40.0) 6(40.0)	0.124
**Adjuvant therapy**	No treatment Adjuvant CTx alone Adjuvant CTx and RTx Adjuvant RTx alone	145 31 18 8	81(75.0) 16(14.8) 8(7.4) 3(2.8)	55(69.6) 12(15.2) 8(10.1) 4(5.1)	9(60.0) 3(20.0) 2(13.3) 1(6.7)	0.883*
**Treatment after relapse (n=82)**	CTx alone RTx alone CCRT Re-operation BSC	31 9 12 2 28	18(45.0) 2(5.0) 7(17.5) 1(2.5) 12(30.0)	10(30.3) 5(15.2) 4(12.1) 1(3.0) 13(39.4)	3(33.3) 2(22.2) 1(11.1) 0(0.0) 3(33.3)	0.700^b^

Abbreviations: PLT, platelet; PLR, platelet-to-lymphocyte ratio; SqCC, squamous cell carcinoma; Non-SqCC, non-squamous cell carcinoma; ECOG PS, Eastern Cooperative Oncology Group Performance Status; op, operation; CTx, chemotherapy; RTx, radiotherapy; CCRT, concurrent chemoradiotherapy; BSC, best supportive care

a Including adenocarcinoma, large cell carcinoma, bronchioalveolar carcinoma, non-small cell carcinoma.

b Comparison between patients treated with any salvage therapy and with BSC alone.

* By Fisher's exact test

### Associations of the PLT, PLR, and PLT-PLR score with survival

Both thrombocytosis and a high PLR were significantly associated with poor prognosis in the patients. Patients with thrombocytosis had a shorter OS than that of those without thrombocytosis (79.737 mo vs. 12.715 mo, *p* < 0.001). Patients with a high PLR also had a worse OS than that of those with a low PLR (not reached vs. 38.500 mo, *p* = 0.022).

**Figure 1:**
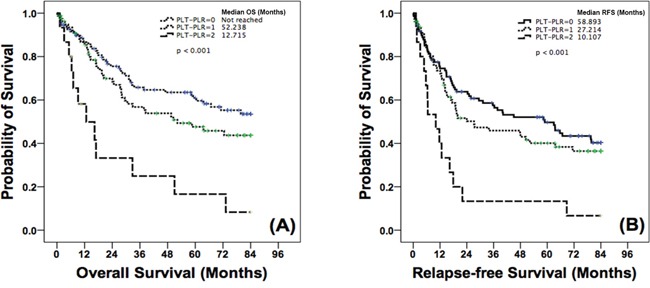
Kaplan-Meier curves for A. OS and B. RFS according to PLT-PLR

Given the significant associations of the PLT and PLR with survival of the patients, we analyzed the association between the PLT-PLR score and patient prognosis. Patients with a PLT-PLR score of 2 had a significantly poorer median OS [12.715 mo; 95% confidence interval (CI) 1.215-24.215] when compared with patients with a PLT-PLR score of 1 (52.238 mo; 95% CI 17.062-87.414, *p* = 0.002 vs. a score of 2) or 0 (not reached, *p* < 0.001 vs. a score of 2; Figure 1A). Relapse-free survival (RFS) was also significantly decreased in patients with a PLT-PLR score of 2 (10.107 mo; 95% CI 3.388-16.826) when compared with patient with a PLT-PLR score of 1 (27.214 mo; 95% CI 0-56.253, *p* = 0.002 vs. a score of 2) or 0 (58.893 mo; 95% CI 32.938-84.848, *p* < 0.001 vs. a score of 2; Figure 1B).

Salvage therapy after relapse did not affect the clinical outcomes of the patients in this study. In 82 patients who relapsed after surgery, there were no significant differences in OS (*p* = 0.777) or RFS (*p* = 0.101) between patients treated with any salvage therapy and those treated with BSC alone. Moreover, the proportion of patients treated with salvage therapy did not differ according to the PLT-PLR score (Table 1).

### Cox regression analysis

Univariate and multivariate analyses for survival are shown in Table 2. Univariate analysis identified male sex, age ≥ 65, SqCC, stage III disease, and a PLT-PLR score of 2 as prognostic factors for poor OS, whereas an Eastern Cooperative Oncology Group Performance Status (ECOG PS) of 1 (vs. 0), stage III disease, and a PLT-PLR score of 2 were associated with poor RFS. Multivariate analysis identified a PLT-PLR score of 2 (hazard ratio (HR) 3.473; 95% CI 1.765-6.835, *p* < 0.001) as an independent poor prognostic factor for OS. With respect to RFS, a PLT-PLR score of 2 (HR 2.286; 95% CI 1.243-4.206, *p* = 0.008) was also an independent poor prognostic factor in the multivariate analysis. Harrell's C-index of the Cox model that included the PLT-PLR score (OS 0.653; RFS 0.642) was higher than that in the model that did not include the PLT-PLR score (OS 0.630; RFS 0.627).

**Table 2:** Cox proportional regression model for OS and RFS

Variables	OS						RFS					
	Univariate			Multivariate			Univariate			Multivariate		
	HR	95% CI	*P*	HR	95% CI	*P*	HR	95% CI	*P*	HR	95% CI	*P*
**Sex**												
Male	Reference	0.247-0.919	0.027	Reference	0.331-1.296	0.224	Reference	0.435-1.216	0.224			
Female	0.477			0.655			0.727					
**Age (yr)**												
< 65	Reference	1.090-2.545	0.018	Reference	1.322-3.143	0.001	Reference	0.909-1.905	0.146			
≥ 65	1.666			2.038			1.316					
**Smoking**												
Never smoker	Reference	0.624-1.465	0.956				Reference	0.657-1.409	0.842			
Current or former smoker	0.835						0.962					
**Histology**												
SqCC	Reference	0.400-0.969	0.036	Reference	0.571-1.456	0.698	Reference	0.505-1.087	0.126			
Non-SqCC	0.623			0.911			0.741					
**ECOG PS**												
0	Reference	0.932-2.146	0.104				Reference	1.041-2.187	0.030	Reference	0.985-2.113	0.060
1	1.414						1.509			1.443		
**Surgery**												
Lobectomy and others	Reference	0.660-1.993	0.626				Reference	0.849-2.182	0.201			
Pneumonectomy	1.147						1.361					
**TNM stage**												
pI	Reference			Reference			Reference			Reference		
pII	1.439	0.922-2.246	0.109	1.400	0.887-2.210	0.149	1.636	1.096-2.442	0.016	1.628	1.086-2.438	0.018
pIII	2.260	1.318-3.876	0.003	2.222	1.248-3.954	0.007	2.950	1.812-4.804	< 0.001	2.350	1.394-3.962	0.001
**PLT-PLR**												
0	Reference			Reference			Reference			Reference		
1	1.360	0.882-2.099	0.148	1.356	0.886-2.075	0.161	1.204	0.818-1.774	0.310	1.248	0.853-1.828	0.254
2	3.787	1.986-7.220	< 0.001	3.473	1.765-6.835	< 0.001	3.087	1.712-5.565	< 0.001	2.286	1.243-4.206	0.008

Abbreviation: OS, overall survival; RFS, relapse-free survival; HR, hazard ratio; 95% CI, 95% confidence interval; SqCC, squamous cell carcinoma; Non-SqCC, non-squamous cell carcinoma; ECOG PS, Eastern Cooperative Oncology Group Performance Status; PLT, platelet; PLR, platelet-to-lymphocyte ratio

### Subgroup analysis

We investigated the prognostic value of PLT-PLR relative to TNM stage, age, smoking status, sex, and histology (Table 3). A strong association between PLT-PLR and OS was found irrespective of age (*p* = 0.013 for < 65; *p* < 0.001 for ≥ 65), smoking status (never smoker, *p* = 0.007; current or ex-smoker, *p* = 0.002), and sex (male, *p* = 0.002; female; *p* = 0.001). The low PLT-PLR group (score 0 or 1) also showed a better OS relative to the high PLT-PLR group (score 2) for stage I/II disease (*p* = 0.002) and SqCC (*p* < 0.001). Furthermore, a strong association between PLT-PLR and RFS was also found for stage I/II disease (*p* = 0.003), age ≥ 65 (*p* < 0.001), current or ex-smoker (*p* = 0.002), male sex (*p* = 0.002), and SqCC (*p* < 0.001).

**Table 3:** Subgroup analysis for OS and RFS according to PLT-PLR score

	PLT-PLR	N (%)	OS, months			RFS, months		
			Median (SD)	95% CI	*P*	Median (SD)	95% CI	*P*
**Stage**								
pI and pII	0	89(53.9)	61.014(3.377)	54.395-67.632	0.002	53.384(3.619)	46.291-60.477	0.003
	1	67(40.6)	51.197(4.062)	43.236-59.158		43.905(4.290)	35.497-52.313	
	2	9(5.5)	27.695(9.395)	9.282-46.108		18.370(8.079)	2.535-34.205	
pIII	0	19(51.4)	37.717(18.733)	1.001-74.433	0.221	11.663(4.451)	2.939-20.388	0.724
	1	12(32.4)	38.500(17.175)	4.837-72.163		12.057(5.620)	1.042-23.073	
	2	6(16.2)	9.166(3.106)	3.078-15.255		6.505(2.286)	2.024-10.986	
**Age (yr)**								
< 65	0	50(54.9)	64.991(4.407)	56.353-73.628	0.013	53.127(5.051)	43.227-63.028	0.207
	1	34(37.4)	55.156(6.092)	43.216-67.096		44.467(6.610)	31.512-57.422	
	2	7(7.7)	37.428(11.515)	14.858-59.998		30.329(11.378)	8.027-52.630	
≥ 65	0	58(52.3)	66.793(23.153)	21.412-112.173	< 0.001	40.214(18.758)	3.449-76.980	< 0.001
	1	45(40.5)	50.464(17.928)	15.325-85.604		27.214(18.203)	0.000-62.891	
	2	8(7.2)	9.166(3.505)	2.297-16.036		6.374(1.165)	4.090-8.658	
**Smoking**								
Never smoker	0	34(50.0)	58.563(5.396)	47.988-69.138	0.007	47.126(5.770)	35.816-58.436	0.108
	1	32(47.1)	51.704(6.285)	39.386-64.022		45.813(6.529)	33.016-58.609	
	2	2(2.9)	9.544(3.170)	3.330-15.758		9.544(3.170)	3.330-15.758	
Current or Ex-smoker	0	74(55.2)	58.121(3.960)	50.359-65.884	0.002	49.736(4.190)	41.524-57.948	0.002
	1	47(35.1)	50.277(4.826)	40.818-59.736		40.399(5.081)	30.441-50.356	
	2	13(9.7)	29.472(8.539)	12.737-46.208		19.524(6.987)	5.830-33.218	
**Sex**								
Male	0	88(52.1)	55.027(3.673)	47.829-62.226	0.002	47.102(3.833)	39.589-54.615	0.002
	1	67(39.6)	49.096(4.189)	40.885-57.307		41.603(4.378)	33.023-50.184	
	2	14(8.3)	27.212(8.136)	11.265-43.159		18.658(6.545)	5.829-31.486	
Female	0	20(60.6)	71.272(5.302)	60.880-81.664	0.001	65.676(2.783)	60.220-71.131	0.099
	1	12(36.4)	61.945(8.848)	44.603-79.286		33.857		
	2	1(3.0)	16.953			11.696		
**Histology**								
SqCC	0	61(48.8)	57.701(4.282)	49.308-66.095	< 0.001	50.523(4.510)	41.684-59.362	< 0.001
	1	53(42.4)	43.592(4.575)	34.626-52.559		34.520(4.601)	25.501-43.538	
	2	11(8.8)	20.987(7.558)	6.172-35.802		14.913(5.573)	3.990-25.836	
Non-SqCC	0	47(61.0)	58.616(4.824)	49.161-68.071	0.096	46.457(5.126)	36.411-56.503	0.098
	1	26(33.8)	66.095(5.900)	54.531-77.659		59.314(6.731)	46.121-72.507	
	2	4(5.2)	35.368(17.107)	1.839-68.897		27.215(16.922)	0.000-60.382	

Abbreviations: PLT, platelet; PLR, platelet-to-lymphocyte ratio; SqCC, squamous cell carcinoma; Non-SqCC, non-squamous cell carcinoma

## DISCUSSION

In the present study, we assessed the prognostic role of the PLT-PLR score in NSCLC patients who underwent curative surgical resection. Patients with a PLT-PLR score of 2 had very poor prognoses, with a median OS of only 12 months, which is similar to that reported in advanced NSCLC patients treated with palliative platinum-doublet chemotherapy [16]. The prognostic value of the PLT-PLR score was independent of age, sex, histology, and tumor stage. Furthermore, the prognosis of the patients worsened as the PLT-PLR score increased from 0 to 1 to 2. A PLT-PLR score of 1 indicates either the absence of thrombocytosis with relative lymphopenia (PLT ≤ 450 × 10^3^/μL and PLR > 160) or the absence of relative lymphopenia with thrombocytosis (PLT > 450 × 10^3^/μL and PLR ≤ 160), whereas PLT-PLR scores of 0 and 2 indicate the absence and the presence of both thrombocytosis and relative lymphopenia, respectively, in most cases. These findings imply that the PLT-PLR score reflects the prognostic roles of platelets and lymphocytes more specifically compared with the PLR alone, and that thrombocytosis and lymphopenia may contribute equally to the poor prognosis of patients with higher PLT-PLR scores.

There is much evidence that suggests an association among platelets, lymphocytes, and tumor biology. Under normal conditions, platelets act as an important modulator of numerous physiological processes, including immune function, wound healing, and angiogenesis, as well as mediation of thrombus formation [17, 18]. However, tumor-associated angiogenesis through the release of vascular endothelial growth factor from megakaryocytes has been shown to promote tumor growth and metastasis [7, 19, 20]. Tumor cell arrest within the organ vasculature, a key process for hematogenous metastasis, is promoted by platelets [21]. Platelets promote tumor invasion by causing the breakdown of the vessel basement membrane via the release of proteolytic enzymes such as metalloproteinase-9 [7, 22]. Furthermore, malignant tumor cells have the ability to aggregate platelets, resulting in so-called tumor cell-induced platelet aggregation (TCIPA) [23]. TCIPA allows tumor cells to evade immune surveillance and to be protected from physical clearance [23]. In contrast to platelets, lymphocytes have been associated with antitumor effects, based on the concept of ‘cancer immunosurveillance’. T cells secrete cytokines and induce acute inflammation, which result in a tumor environment that enhances cytotoxic T cells and tissue destruction [24]. Natural killer (NK) cells also have antitumor effects through direct cytolytic activity and the production of cytokines [25, 26]. In various types of cancer, increased infiltration of tumor-infiltrating lymphocytes (TILs) is associated with a good prognosis and favorable responses to anticancer therapy [27–29]. This evidence, which is in opposition to the effects of platelets and lymphocytes on tumor biology, supports our suggestion that a marker incorporating both values is needed for a more reliable prediction of prognosis in cancer patients.

Several lines of evidence suggest a relationship between PLR and survival across several types of cancer. A recent Chinese study examining pretreatment PLR scores in 210 advanced NSCLC patients suggested the PLR may be useful to predict disease outcomes and the response to first-line chemotherapy [10]. In this study, an elevated PLR was associated with a 2-fold risk of death and a 4.5-fold risk of early progression. Similar findings were observed in a previous study that evaluated 372 stage II-III colon cancer patients who underwent surgery [30]. In that study, the patients with an elevated PLR had a 65% increased risk of recurrence. A large Austrian study examining 793 non-metastatic breast cancer patients showed that an elevated preoperative PLR increases the risk of death two-fold [11]. Taken together, these data suggest that an elevated PLR is significantly related to poor prognosis in patients with cancer. In addition to the clinical significance of PLR, its high accessibility makes this marker useful in clinical practice. Complete blood counts are obtained in all patients undergoing planned surgery, meaning that the PLR can be measured easily in nearly all patients, thereby eliminating the need for additional tests to obtain this marker. Moreover, this approach is also faster and cheaper than other conventional markers such as serum CEA, CA 19-9, SCC, NSE, and CYFRA 21-1. However, measuring the PLR alone has a disadvantage in some cases. For example, in the presence of severe lymphopenia, the PLR may increase even in patients without thrombocytosis. In the presence of lymphocytosis, the PLR may decrease even in patients with thrombocytosis. Instead, as described above, the PLT-PLR score can discriminate these ambiguous cases by appointing a score of 1, which suggests an intermediate prognosis.

As with all studies, this work has several limitations, which should be taken into consideration. First, the sample size was relatively small for generalizing the clinical significance of the PLT-PLR in resectable NSCLC. Moreover, among the 202 patients enrolled, the number of patients with a PLT-PLR score of 2, which is associated with the worst prognosis, was only 15 (7.4%). Therefore, our data must be regarded as preliminary. In particular, the subgroup analysis (Table 3) should be interpreted with caution. Confirmation of the present results in an independent data set is imperative for drawing firm conclusions about the role of PLT-PLR for NSCLC prognosis. Second, the cut-off values used for each marker comprising the PLT-PLR score were not confirmative. The cut-off value for the PLR was determined using a minimal *p*-value approach, which leads to inflation of the type I error rate [31]. The PLT cut-off value of 450 × 10^3^/μL is commonly used in clinical practice. Although the prognosis of the patients was significantly influenced by either the PLT or PLR alone, further analyses are necessary to establish cut-off values for each marker.

In conclusion, based on the PLT-PLR scoring system, patients with resectable NSCLC could be classified into three groups with different prognoses in this study. This platelet- and lymphocyte-based prognostic model may be useful for predicting postoperative outcomes and for individualizing postoperative management plans in patients with surgically resectable NSCLC. A large prospective study is needed to properly validate our findings.

## MATERIALS AND METHODS

### Study population

We retrospectively reviewed all patients histologically confirmed for NSCLC who were treated by surgical resection at Gyeongsang National University Hospital Regional Cancer Center (Jinju, Korea) and Samsung Changwon Hospital (Changwon, Korea) between January 2002 and December 2007. Inclusion criteria were age ≥ 18 years, an ECOG PS of 0 or 1, and adequate hematologic, liver, and kidney function. Patients were excluded if they exhibited clinical evidence of infection or other inflammatory conditions, or were treated previously with neoadjuvant chemotherapy or radiotherapy. Our Institutional Review Board approved this retrospective study (2012-SCMC-034-00) and waived the requirement for informed consent. Baseline characteristics including demographics, smoking status, performance status, and medical history were collected using an electronic medical record system. Complete blood cell counts with differential counts at diagnosis were evaluated.

### Diagnostic criteria

The PLR was defined as the absolute PLT divided by the absolute lymphocyte count. The optimal cut-off value for PLR was determined using a minimal *p*-value approach. PLR values were categorized into two groups: < 160 and ≥ 160. A PLT > 450 × 10^3^/μL was defined as thrombocytosis. Patients were assigned a PLT-PLR score of 0, 1, or 2 based on the presence of thrombocytosis, an elevated PLR (> 160), or both, as follows: patients with both thrombocytosis and an elevated PLR were assigned a score of 2, and patients with either or neither were assigned a score of 1 or 0, respectively.

### Statistical analysis

The association between clinicopathological parameters and the PLT-PLR was analyzed using the χ^2^ or Fisher's exact test, as appropriate. OS was calculated from the date of diagnosis to the date of death from any cause or the date of the last follow-up observation. RFS was calculated from the date of diagnosis to the date of recurrence or the date of death from any cause. Patients who did not die during the course of follow-up were censored at the date last seen alive. Kaplan-Meier analysis and log rank tests were used to compare the postoperative survival curves between groups. Univariate and multivariate analyses of survival were conducted using the Cox proportional hazards model with the Enter selection method. All variables with *p* < 0.1 in the univariate analyses were included in the multivariate analysis. The predictive ability of the models was evaluated using Harrell's C-index. *P* values < 0.05 were considered statistically significant. All statistical analyses were conducted using SPSS for Windows (ver. 18.0; SPSS Inc., Chicago, IL, USA) and STATA software (ver. 14.0; StataCorp, College Station, TX, USA).
